# Evolutionary morphology of genital spines informed by puncture mechanics

**DOI:** 10.1098/rspb.2025.1698

**Published:** 2025-08-20

**Authors:** Rachel Keeffe, Bingyang Zhang, Philip S. L. Anderson, Patricia L. R. Brennan

**Affiliations:** ^1^ Department of Biology, Mount Holyoke College, South Hadley, MA, USA; ^2^ Department of Biology, Trinity College, Hartford, CT, USA; ^3^ Ingram School of Engineering, Texas State University, San Marcos, TX 78666, USA; ^4^ Department of Evolution, Ecology and Behavior, University of Illinois, Champaign, IL 61801, USA

**Keywords:** instron, compression, hemipenis, reproduction, curvature, fracture

## Abstract

Genital spines are widespread in animals with internal fertilization, and their morphology is diverse. Many hypotheses have been proposed to explain their presence, yet their function has not been tested in most systems. We do not yet have a framework to characterize their morphology and potential function, even to determine if they puncture the vaginal tract (as is often assumed). Here, we examine the morphospace and puncturing ability of genital spines by testing different 3D-printed spines from a diverse sample of μCT-scanned snake hemipenes, which are among the most morphologically variable intromittent genitalia in vertebrates. We performed serial compression tests into polydimethylsiloxane silicone polymer at increments of 10° until puncture was impossible. We found that the range of successful puncture angles differed based on spine curvature and tip sharpness, suggesting that some spines are built to puncture at almost every angle of approach, while others are extremely unlikely to puncture vaginal tissues at all. These results provide a framework for continued study and characterization of the shape and performance of genital spines in other animal groups and help to refine functional hypotheses in the context of copulatory interactions.

## 1. Introduction

Spines and hooks are biological structures that generally function to increase friction or achieve puncture into a substrate, and their morphological diversity is associated with their varied functions that include predation, defence, locomotion and reproduction [1–4]. The evolution of puncture and grasping in reproductive systems seems to be widespread as revealed by the common occurrence of spines and hooks in copulatory structures in groups as diverse as insects, arachnids, fishes, frogs, squamates and mammals [4–6]. The function of puncture and grasping in these systems has been hypothesized to be driven by sexual selection and/or sexual conflict. Examples of this include harming the female reproductive tract to prevent quick remating [7], perforating the female reproductive tract to allow seminal fluid to enter the female coelom [8], grasping the female to prolong or facilitate copulation [9], stimulating the female to increase paternity success [10] and removing copulatory plugs [11]. Natural selection could also play a role (e.g. by providing sensory feedback to the male to allow ejaculation to take place [12]). Despite their frequent occurrence in nature and potentially diverse functions, the morphological diversity and performance of genital spines and hooks in perforating and grasping have not been examined in detail.

To examine the puncturing performance of genital spines, one clade of particular interest is snakes, a group with an extreme diversity of genital morphology that includes spines and hooks of varying shape, size and number. Snakes have paired intromittent structures called hemipenes that can be covered in complex external features like chalices, flounces and calcified spines and hooks [13]. Previous studies have suggested that hemipenial spines help males hold on to females during copulation because snakes lack limbs that can aid in grasping [14]. However, this hypothesis has not been tested, and the absence of spines in many limbless squamates suggests that spines are not necessary for successful copulation. The only experimental manipulation of spines in snakes so far has involved the removal of the large basal hook of the hemipenes in red-sided garter snakes (*Thamnophis sirtalis*). These tests determined that this basal hook functions to prolong rather than facilitate copulation, while also showing that the spines penetrate deeply into the vaginal mucosa and cause extensive bleeding [9].

While copulation sometimes requires erection prior to intromission, some species, including snakes, evert their intromittent organs into the vagina. Hemipenes are kept inside the tail in an inverted position with the spines facing the inside of the hemipenis at rest. Eversion causes the spines to rotate around the everting edge of the hemipenis. This rotation, in conjunction with the hemipenis everting out into the vaginal pouch, can engage the spine in the vaginal tissue. It is assumed that puncture of the vagina secures the hemipenis in place for successful copulation [15].

Modern morphological techniques such as automated landmarking software and the growing accessibility of high-resolution μCT scanners have opened the door to begin quantifying the rich biological morphospace of genital spines, as well as the relationship between spine shape and function. Here, we quantify for the first time the morphological variation and the puncture mechanics of different genital spine shapes obtained from a sample of snakes. We assess how spine shape is related to puncture mechanics and describe possible functional explanations about spine diversity observed in nature. These questions will improve our understanding of the evolutionary forces driving genital evolution and will help provide a broader context for the role of spines in other animals with intromittent organ spines such as mammals and insects.

## 2. Material and methods

### (a) Specimen preparation

We inflated and collected one hemipenis (following general methods described in [16,17]) from 11 snake species belonging to four different families known to bear spines on the hemipenes (table 1): Elapidae (*Acanthophis rugosus*), Viperidae (*Crotalus atrox*, *Crotalus viridis*), Lamprophiidae (*Mehelya crossi*) and Colubridae (*Nerodia fasciata*, *Nerodia rhombifer*, *Nerodia sipedon*, *Opheodrys aestivus*, *Pantherophis alleghaniensis* and *Pantherophis guttatus*). Frozen snakes were sourced from donations from collaborators (see Acknowledgements) or otherwise purchased from online retailers (ReptilesNCritters.com, Backwater Reptiles Inc.) live and then humanely euthanized with tricaine methanesulfonate [18].

**Table 1.** Species sampled, snout–vent length (SVL) in mm and hemipenial spine properties. Spine count is approximate, with asterisks representing specimens that were vastly underestimated due to spine proximity disrupting the islands function in 3D Slicer.

family	species	SVL (mm)	spine count	largest spine volume (mm^3^)	highest structural curvature (°)
Colubridae	*Ahaetulla nasuta*	628	489	0.02372	45.79
Colubridae	*Nerodia fasciata*	516	955	0.26906	97.69
Colubridae	*Nerodia rhombifer*	664	2698*	1.04361	54.58
Colubridae	*Nerodia sipedon*	487	1292*	0.24166	109.69
Colubridae	*Opheodrys aestivus*	NA	769	0.11474	73.68
Colubridae	*Pantherophis alleghaniensis*	1222	469	0.03729	26.56
Colubridae	*Pantherophis guttatus*	805	337	0.00629	36.7
Elapidae	*Acanthophis rugosus*	480	419	0.00794	24.55
Lamprophiidae	*Mehelya crossi*	1055	975	0.01896	76.01
Viperidae	*Crotalus atrox*	827	301	0.03822	16.89
Viperidae	*Crotalus viridis*	763	308	0.03822	28.89

All specimens were fixed in 10% formalin and then CT scanned with a μCT Bruker Skyscan 1276. Scanning parameters were as follows: voltage = 55 kV, current = 200 µA, filter = aluminium 0.25 mm. The scan data were reconstructed with NRecon v. 1.7.4.2 and exported to Slicer v. 5.2.2 via the SlicerMorph extension for segmentation [19,20].

**Figure 1.**
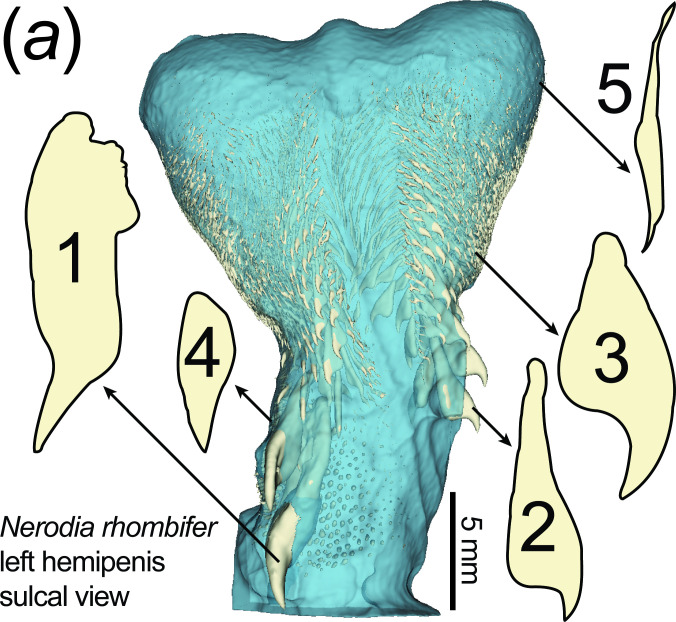
Three-dimensional rendering of the left hemipenis of *Nerodia rhombifer* in sulcal view, highlighting the morphological variation of hemipenial spines. Soft tissue is rendered in semi-transparent light blue, and calcified spines are rendered in opaque, pale yellow. Silhouettes of five spines are illustrated around the hemipenis, with an arrow pointing to their approximate position on the shaft. Silhouette numbers correspond to the IDs used in the manuscript. The scale bar applies only to the hemipenis; spine silhouettes are not shown to scale.

All spines were segmented via contrast thresholding in Slicer (parameters recorded in electronic supplementary material, table S1). A single hemipenis can have thousands of individual spines (e.g. *N. rhombifer* had more than 2698 individual spine elements calculated via the islands tool in Slicer v. 5.2.2; see figure 1 and table 1), so not all could be tested, but we isolated the largest spine by volume and exported it as a mesh file. In some hemipenes, one spine was clearly the largest but in others there were several large spines of similar volume. We also isolated and exported any other spines that differed markedly in shape from the largest spine in order to sample spine shapes broadly. We considered four binary categories to determine whether a shape was ‘markedly different’ and whether the spine was elongate or short (e.g. short spines having an aspect ratio approximately over 50%), hooked or not-hooked, straight or wavy, and conical or laterally compressed. If a spine with a unique combination of these traits was present, it was included in the sample from that individual. In total, 41 spines were chosen for the analysis (electronic supplementary material, table S1). As a result of using this method, we captured the most distinct shapes rather than a sample of all possible shapes.

### (b) Three-dimensional shape analysis

We analysed the shape of the 41 distinct spines using a parallelized version of automated landmarking software Auto3DGM [21,22]. Initial alignment was done with 200 landmarks and final alignment with 3000 landmarks. Aligned landmark data were imported in R (v. 4.4.1) for analysis [23]. We ran a generalized procrustes analysis to move data into the same shape space and then conducted a principal component analysis on the results using the R package *geomorph* v. 4.0.8 [24,25]. We did this in order to characterize the shape variation in a quantitative way.

To determine which spines to use for mechanical testing, we then created a subsample (*n* = 13) along the entirety of principal component 1 (PC1), so as to represent the range of morphotypes as well as one specimen from each of the four snake families. We included all distinct spine types from *N. rhombifer*, as a representative example of shape diversity in a single hemipenis (figure 1).

We also ran a phylomorphospace using a recent tree of caenophidian snakes [26] and the R package *phytools* v. 2.3-0 [27] to investigate what role, if any, phylogenetic relationships played in our dataset (electronic supplementary material, figure S1). This phylomorphospace included only the largest hemipenial spines (by volume) of each individual.

### (c) Tool and substrate preparation

The 3D mesh file for each subsampled spine was loaded into Blender v. 3.2.2 [28] and scaled to the same dimensions (longest axis = 3.22 cm). We 3D-printed the scaled spines using Clear V4 on a Form 2 printer (Formlabs, Somerville, MA) with a Z-resolution of approx. 0.1 mm. Support structures were placed along the anchoring portion of the spine to prevent imperfections at the functional tip of each spine. The models were cleaned in isopropyl alcohol for 20 min and then cured on a rotating stand beneath ultraviolet light for 1 h. The tensile modulus of the cured Clear v. 4 resin is 2.8 GPa. The 3D printing supports were removed with wire cutters and all spines were photographed prior to testing with a Leica microscope camera (M205C; Leica Camera AG, Wetzlar, Germany). We prepared 90 g (approx. 5 cm^3^) cubes of polydimethylsiloxane (PDMS) silicone polymer using the Sylgard 184 (Dow Chemical Company, Midland, MI) commercial kit as received at a 10 : 1 ratio of base to curing agent for our puncture substrate. PDMS cubes were cured at 70°C for 2 h. PDMS cubes were prepared to have a modulus that mimicked that of human and pig skin (approx. 1 MPa [29,30]).

### (d) Puncture performance

**Figure 2.**
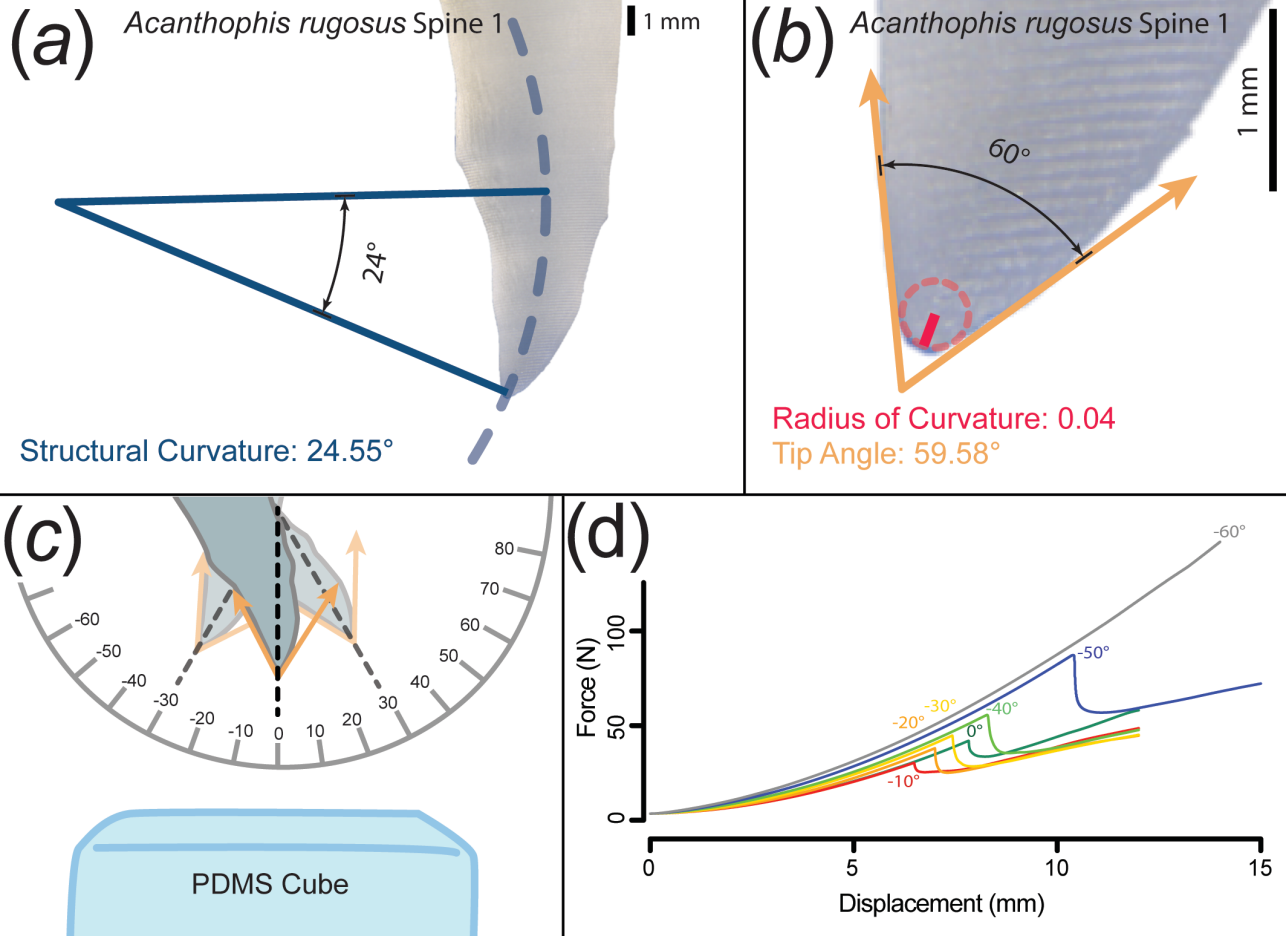
(a) Diagram of structural curvature calculation. The blue dotted line is a circle that maximally bisects the spine, and the solid blue lines represent the structural curvature as measured from the tip to where the fitted circle no longer bisects the spine. (b) Diagram of radius of curvature and tip angle. The dotted red line represents the circle fit to the tip of the spine, the solid red line indicates the radius of curvature and the solid orange lines represent the tip angle. (c) Diagram of experimental set-up for puncture tests. The solid orange lines represent the tip angle. (d) An example of raw output from those tests, where a sudden drop in force indicates a successful puncture event.

We modified a protocol used previously to test puncture in other systems [31–33]. We used a Universal Testing System (Instron 3344; Instron) with a 500 N loading cell and clamps (Screw Side Action Tensile Grips; Instron) to perform all compression tests and record the associated force–displacement curves. Puncture was determined based on sharp drops in these curves (figure 2*d*). For each test, the travel limit was set between 15 and 5 mm and the compression rate was set to 10 mm min^−1^. We tested each tool point-down, with the vertex of the tip angle pointing straight down towards the PDMS substrate cube, and then conducted subsequent tests at increments of 10° in either direction (along the plane bisecting the spine perpendicular to the hemipenial surface; hereafter, local sagittal plane) until the tool no longer achieved puncture. Each tool and angle combination was replicated three times. See figure 2 for an illustration of the experimental design and raw data output examples. One spine, NERH_5, broke during testing and was excluded from all puncture performance analyses.

Because spines probably change their angle as the intromittent organ opens, everts or inflates, we tested puncturing performance at several angles (10° increments from 0°) rather than a single angle—which is a more typical method to test puncturing performance in other systems [34]. We recorded the number of different angles at which each spine could puncture and the average amount of force (across the three replicate trials) needed to achieve puncture for each of those angles. We then calculated the average force required to achieve puncture across all angles tested, as well as the minimum amount of force needed to puncture across all tests for each spine.

### (e) Assessing tool properties

The radius of curvature (i.e. the radius of the circular arc that best fits the tip of the tool [31]), tip angle (i.e. the angle formed by the tapering of the tool [31]) and structural curvature (i.e. a geometrical model where a circle is fit to the curvature of the entire tool [35]) were calculated for each spine in ImageJ (v. 1.54 g [36]), from screenshots of the scaled three-dimensional meshes in Blender, viewed perpendicular to the local sagittal plane. Radius of curvature was recorded in pixels, but it should be noted that all spines were scaled to the same length and this measure no longer reflects biological size.

To assess which tool properties, if any, correlated with puncture performance, we ran a series of pairwise linear models in R between average puncture force and the following variables: radius of curvature, tip angle, PC1 score (a proxy for shape), volume and structural curvature. We repeated those linear models replacing average puncture force with puncture range, as calculated as the range of angles where the tool achieved puncture, and also lowest force required to achieve puncture across all trials.

To test which properties (if any) correlated with spine shape, we evaluated the relationship between shape and the following variables using the procD.lm function in *geomorph*: species, family, SVL, structural curvature, tip angle and radius of curvature.

## 3. Results

**Figure 3.**
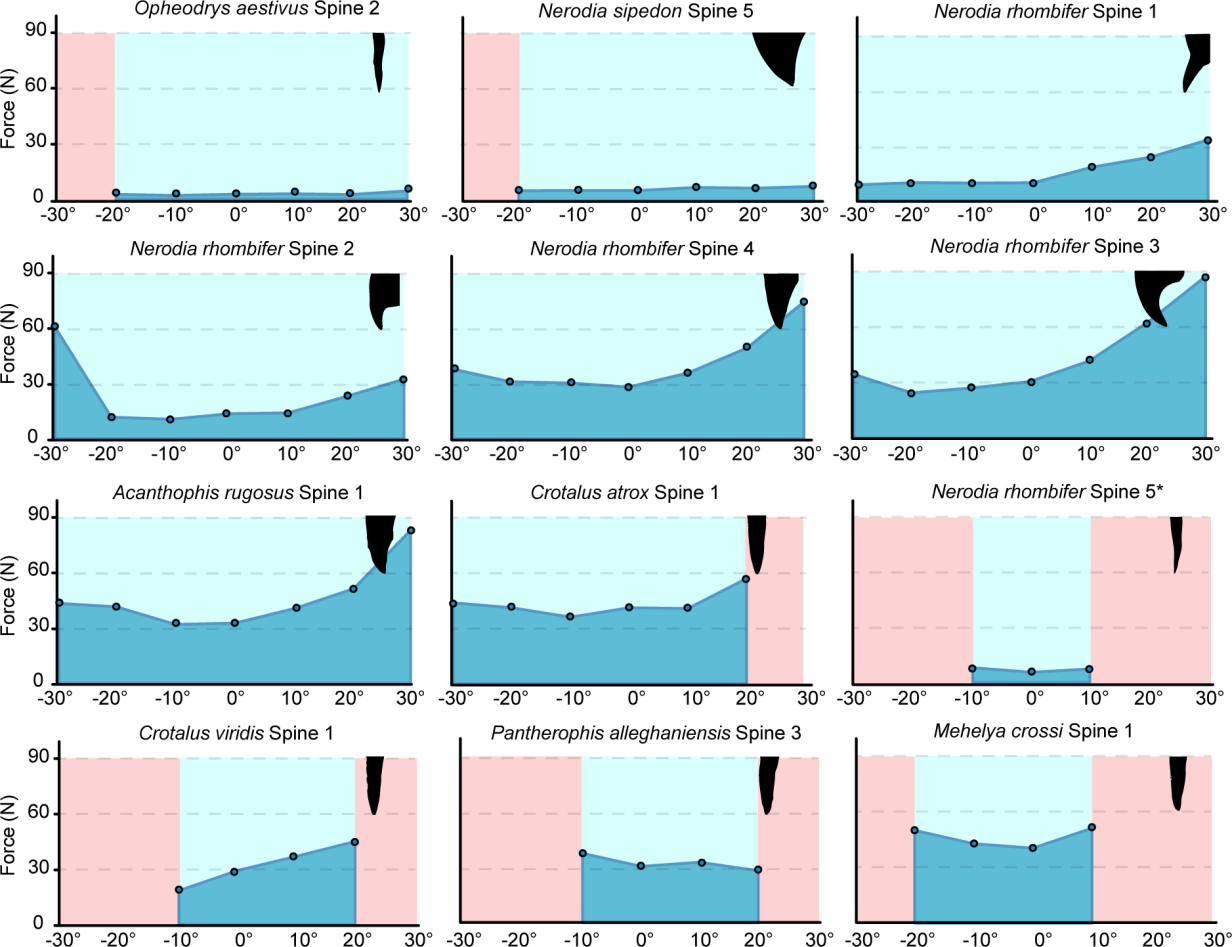
Average force to puncture plotted against different puncture angles (60°). Successful puncture angles are shaded in light blue, and unsuccessful angles are shaded in light red. A silhouette of each spine tip is shown for each panel. An asterisk marks the one tool that broke during testing.

**Figure 4.**
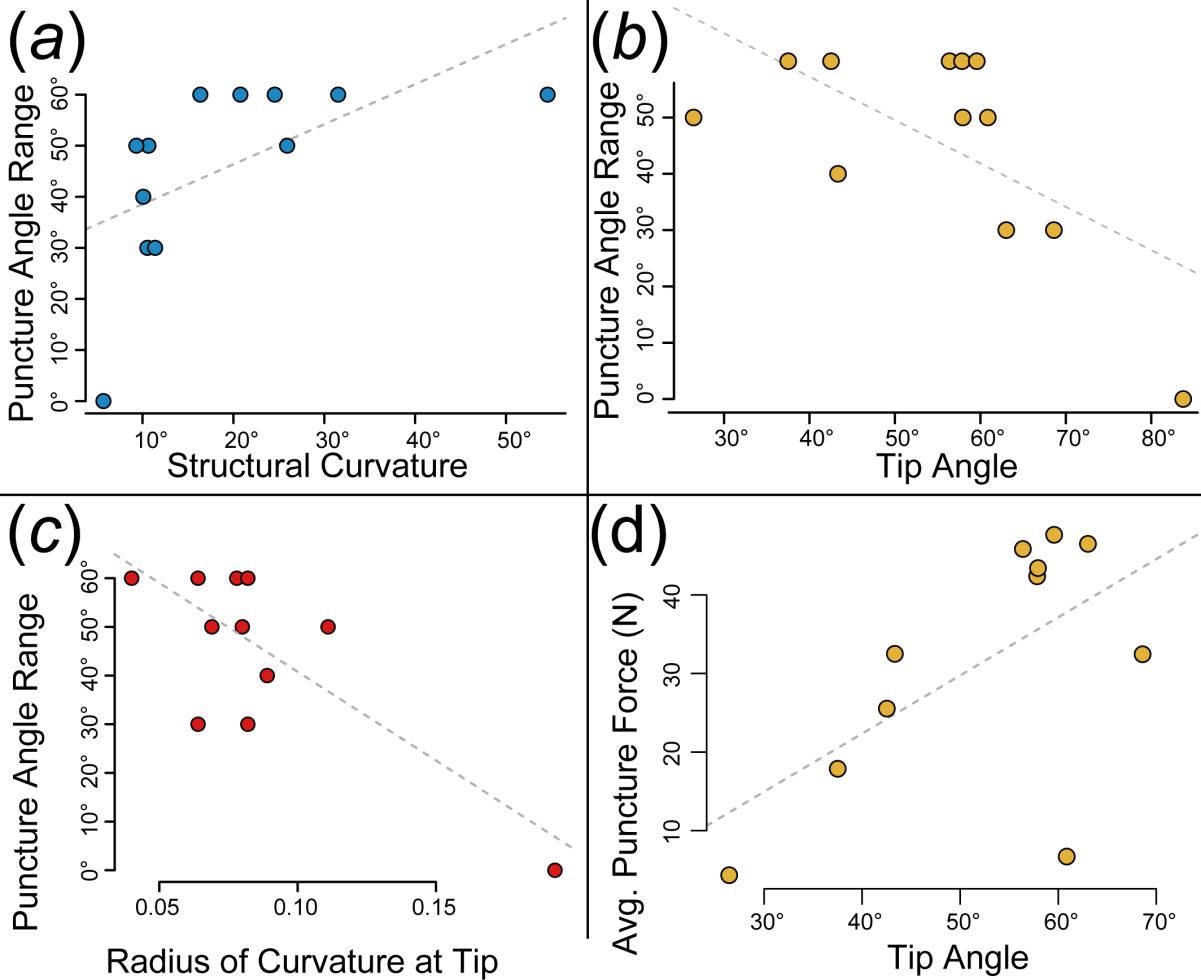
A regression between the range of angles at which puncture was possible and (*a*) the structural curvature of the tool, (*b*) tip angle and (*c*) radius of curvature. (*d*) A regression between the average force to puncture and the tip angle. In all panels, a grey dotted line indicates the regression line.

We found that hemipenial spines are incredibly diverse in both morphology and puncture performance. Spines varied in 3D shape, sharpness, curvature and volume, with some spines incredibly sharp and needle-like while others were deeply hooked and more blunt. Spines varied in puncture performance, with some spines able to puncture across a 60° range with low forces needed to pierce the substrate while others were not capable of puncture at all (figure 3). Tip sharpness and structural curvature significantly predicted puncture angle range (significant relationships plotted in figure 4). Tip angle was marginally significant in predicting average force to achieve puncture (electronic supplementary material, table S2).

### (a) Spine morphology

CT scanning and segmentation are extremely effective in determining spine shape, size and distribution in snakes. Spines are attached to the skin of the hemipenis with an anchoring region deep into the epidermal tissue, while the typically sharp end of the spine is exposed on the surface (figure 1). Spines are highly variable in shape and size, can appear anywhere on the hemipenis except within the *sulcus spermaticus* and can be distributed in varying densities (e.g. *N. rhombifer*; figure 1). All species had hundreds to thousands of calcified structures on the surface of the hemipenis. The volume of the largest spine in each species varied from 1.043 mm^3^ for *N. rhombifer* (23% of the total volume of all spines, snake 664 mm SVL) to a fraction of that value as in *Pantherophis guttatus* (0.00011 mm^3^, 0.00033% of the total volume of all spines, snake 805 mm SVL; (table 1; electronic supplementary material, table S1).

**Figure 5.**
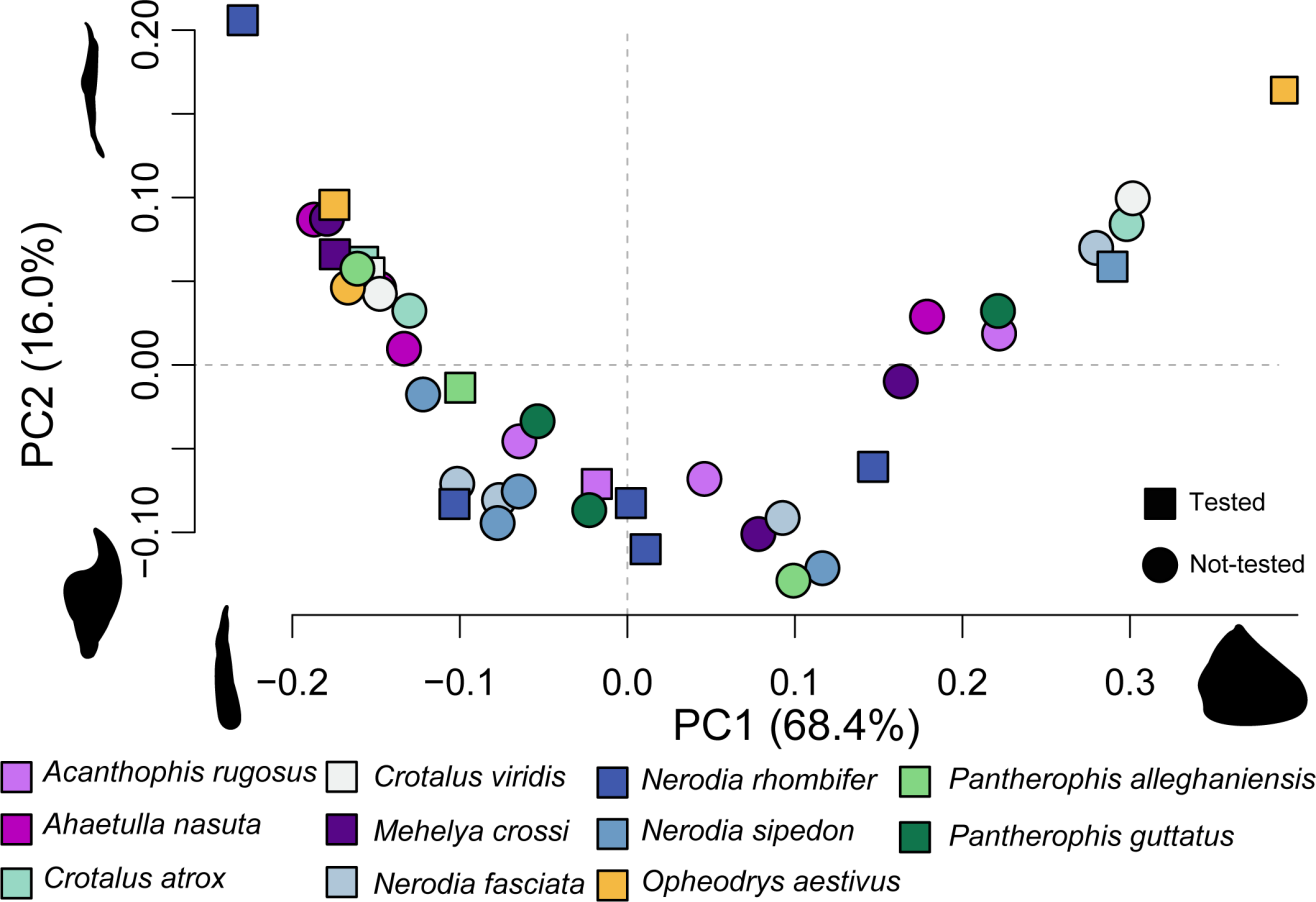
A morphospace of hemipenial spine shape variation along principal components 1 (PC1) and 2 (PC2). Each spine is colour-coded by species. Spines that were tested for puncture performance are indicated with squares; untested spines are indicated with circles.

The 3D geometric morphometric analysis from 41 distinct spine morphologies revealed that spine shape varied primarily between wide conical and needle-like spines. This is represented on PC1, which describes 68.4% of shape variation (figure 5). PC2 describes 16% of shape variation, where positive PC2 values correlate with straight spines and negative values with more curved, hooked spines with a broad base (figure 5). Spine shape was not significantly correlated with any variables tested: species, family, SVL, structural curvature, tip angle or radius of curvature (electronic supplementary material, table S2). Indeed, spines from the same hemipenis often spanned the entire range of morphospace (e.g. *Opheodrys aestivus*; figure 5). Our phylomorphospace confirmed that even when only comparing largest spines across our species, the single family Colubridae dominates spine shape morphospace (electronic supplementary material, figure S1).

The highest spine structural curvature found in each species varied from 109.69° in *N. sipedon* (reflecting a hook-shaped spine) to only 16.89° in *Crotalus atrox* (reflecting a needle-like spine; see table 1). Spine tip properties were also variable, with tip angle spanning from 12.80° in *N. rhombifer* spine 5 reflecting a sharp spine, to 89.12° in *Ahaetulla nasuta* spine 4 reflecting a blunt spine (electronic supplementary material, table S1) and radius of curvature ranging from 0.025 in *N. rhombifer* spine 5 to 0.60 in *Crotalus atrox* spine 3.

### (b) Puncture performance

Puncture performance varied widely across our sample, both within and between species (figure 3; table 2). Indeed, one individual, *Opheodrys aestivus*, had both the best and worst performing hemipenial spines. *Opheodrys aestivus* spine 2 had the lowest force needed to achieve puncture (3.48 N) and could puncture across 50°, and *Opheodrys aestivus* spine 3 could not puncture at all regardless of angle (table 2).

**Table 2.** Spines tested for puncture performance, with species, tip properties (tip angle and radius of curvature), the range of angles for successful puncture, the lowest force at which puncture was achieved and the average force needed to puncture across all angles.

species	spine ID	tip angle (°)	radius of curvature at tip	puncture range (°)	lowest puncture force (N)	average puncture force (N)
*Opheodrys aestivus*	OPAE_2	26.441	0.080	50	3.48	4.32
*Opheodrys aestivus*	OPAE_3	83.735	0.193	0	na	na
*Crotalus viridis*	CRVI_1	68.605	0.082	30	18.38	32.45
*Nerodia rhombifer*	NERH_1	37.512	0.064	60	10.31	17.87
*Nerodia rhombifer*	NERH_2	42.525	0.078	60	12.02	25.53
*Nerodia rhombifer*	NERH_3	56.396	0.082	60	26.16	45.85
*Nerodia rhombifer*	NERH_4	57.850	0.082	60	29.46	42.38
*Nerodia rhombifer*	NERH_5	12.795	0.025	20	5.50	6.90
*Mehelya crossi*	MECR_1	63.031	0.064	30	40.83	46.51
*Crotalus atrox*	CRAT_1	57.938	0.111	50	36.14	43.42
*Nerodia sipedon*	NESI_5	60.870	0.069	50	5.77	6.69
*Acanthophis rugosus*	ACRU_1	59.583	0.040	60	33.16	47.66
*Pantherophis alleghaniensis*	PAAL_3	43.338	0.089	40	28.37	32.50

Of all the variables tested, only radius of curvature, tip angle and structural curvature were significant in predicting puncture angle range (electronic supplementary material, table S2). Only tip angle (marginally) predicted average puncture force (*p* = 0.0520; electronic supplementary material, table S2). These findings suggest that puncture performance is primarily driven by tip sharpness, regardless of other factors like shape, and that hooked spines perform better at a variety of angles. None of our tested variables significantly predicted the lowest force to achieve puncture (electronic supplementary material, table S2). The shape of each spine (as reduced to PC1) had no bearing on average puncture force, puncture angle range or lowest force to achieve puncture.

## 4. Discussion

### (a) Spine morphological variation

We found remarkable intra- and inter-specific variation in the shape, size and number of spines in the species we examined. Future quantification and study can reveal how much of this variation is related to the ontogeny of spine formation or if it is indeed functional.

While we considered all calcified structures in the hemipenis as spines, not all of these structures seem optimized for puncture. In particular, the smaller, conical ‘spines’ have such poor puncture performance (e.g. *Opheodrys aestivus* spine 3) that they were unable to perforate the substrate regardless of angle (although see discussion on scaling effects below). More likely, they may represent the early developmental stage of spines, before they grow sharper and become functional. Little is known about the development of calcified spines, but keratinized spines in mammals are known to begin from a placode that becomes keratinized, and the keratin is constantly replaced [37], likely to keep the spine sharp. Alternatively, these non-puncturing spines could act to increase friction with the female tissue.

Our spine morphospace was dominated by the axis of variation between short conical and needle-like spines, but there was also significant variation in how hooked the spines were and how broad their base was. Our morphospace represents only a fraction of the existing spine shapes (the most distinct shapes) and more work will be required to fully examine morphological variation across snakes.

### (b) Spine function

Hemipenial spines are likely functional elements that evolved for puncturing, independent of phylogeny. The variation we found in our puncture results suggests that some hemipenial spines can only puncture efficiently at a specific angle and remain static (those that are more needle-like), whereas others are able to puncture efficiently over a wider range of angles and potentially propagate a wound (those that are more hook-shaped). Spines with a small radius of curvature and narrow tip angle performed the best in our set-up, which coincides with previous work on other puncture tools in nature [31,32,34]. Notably, spines with high structural curvature were able to puncture at a wider range of angles than straighter spines. This result follows the conclusions of Bar-On [35], where highly curved tools were predicted to have high directional stiffness in a range of angles, as well as those of Zhang *et al.* [33], where structural curvature was found to have minimal impact on puncture performance. Taken together, it is likely then that spines with high structural curvature have a higher load-bearing capacity that reduces the chance of buckling, as well as low directional sensitivity, which enables them to puncture at a wider range of angles. These traits might be advantageous in certain situations such as creating and propagating wounds in vaginal tissue.

In snakes specifically, the largest spine at the base of the hemipenis is often termed the ‘basal hook’ due to this characteristic shape. This spine would be the most effective to grasp the vaginal tissue as the hemipenis everts and the hook changes angle during eversion. Clipping this basal hook in red-sided garter snakes resulted in significantly reduced copulation time and therefore reduced transfer of the sperm due to the deposition of a smaller copulatory plug [9,38]. In the absence of the basal hook, red-sided garter snakes attached to the vaginal wall through the many smaller, more needle-like spines found in the middle of the hemipenial shaft, but this attachment was not sufficient to keep the male attached when the female performed body rolls to terminate copulation [38]. Among non-squamate amniotes, many mammals are also reported to have hook-shaped and needle-like spines in their genitalia that likely function in sexual selection [4].

In addition to grasping the female, spines could also perforate and scrape the vaginal wall to prevent remating (seed beetles [7]), transfer seminal fluid more effectively (drosophila [39]), remove sperm from previous matings (damselflies [40]), or induce ovulation or foster pregnancy (alpaca [41]). The diversity of spine shapes and their different puncturing performance would suggest different functions regionally in a single intromittent organ, and across species. Other small spines that we found at high quantities per unit area (particularly in the apical region of natricine hemipenes, figure 1) also probably do not serve as puncture tools. One of these, *N. rhombifer* spine 5, was so fragile it broke during our testing, even when 3D-printed at scale. In addition, their high density would cause force to spread out more evenly, making it harder to puncture (e.g. as in microneedle arrays [42]). We believe that these areas of small, densely packed ‘spines’ are probably serving to increase friction with the vaginal lumen, which is often coated in mucus.

### (c) Spine evolution

Even though spine size, position and shape are highly diverse, spine configuration appears species specific. For example, some species have uniform spines across the hemipenis (e.g. *Mehelya crossi*), whereas others have clear regionalization of spines organized by shape and size (e.g. *N. rhombifer*; figure 1). Previous work on colubrid snakes supports spines being similar within a species as well [43]. The natricines we tested stood out in having huge, hooked spines in comparison to the modest, needle-like spines of the viperids. If anchoring via puncture was the only selective force, we would not expect to see such a diversity of spine shapes. It is likely that differences in the distribution, size and shape of spines determine their function during copulation, as has been suggested in mammals [4].

It is also likely that the shape and dynamic nature of the vaginal pouch influence which regions of the hemipenis develop spines that are optimized for puncture at specific angles or to achieve a specific amount of friction. In addition, spines may serve to stimulate the female during copulation to increase copulatory success. Females might distinguish between different males and either facilitate or hinder sperm transfer during copulation. We need more data regarding the fit between male and female genitalia to begin teasing apart these complexities.

Understanding the vaginal wall, as the substrate interacting with genital spines, will be necessary to fully understand their function. Currently, we have little data on the modulus and material properties of the vaginas of most species, so we used PDMS cubes that mimicked the modulus of human and pig skin (approx. 1 MPa [29,30]), but the vaginal mucosa and the vaginal wall itself will almost assuredly have different properties that will affect how spines perform. It may be that the vaginal pouch is so elastic or so thick that it could reduce the severity of puncture or even prevent puncture (see discussion of pouch thickness in species with spiny hemipenes in [44,45]). This is the case in seed beetles, where species with the most spiny aedeagus have the thickest vaginal walls, while in bats, the vaginal wall of species with spiny penises have thicker collagen layers than those without [46,47]. Certain features on the inside of the vaginal wall may also impact puncture efficacy, such as folds and lobes, as well as the presence of mucus that is typically abundant in the vagina.

The incredible shape diversity that we report in the performance and shape of hemipenial spines warrants further research into their functional capabilities and their role in the evolution of snake genitalia and our understanding of biomechanical puncture tools.

### (d) Considerations

All of these experimental results need to be interpreted in light of potential scaling issues that are unavoidable when dealing with morphological structures this small. Testing the puncture ability of these spines at their biological size (e.g. 0.54 mm, *Opheodrys aestivus* spine 3) would not be feasible. Therefore, we scaled all of the spines upwards, based on length, to allow for experimental tests to be performed and for us to focus on differences in shape. However, this approach does alter the radius of curvature, one of our measures of sharpness, which is not scale-independent. As such, spines that are scaled up are blunter than they are at their biological size. This means that some of the spines we found unable to puncture in our experiments may well be able to puncture the vaginal tissue at their original sizes, when they have sharper tips. However, this same scaling effect applies to all of the spines examined, meaning the comparisons between spine shapes and species remain meaningful.

Furthermore, the sharpness measure based on tip angle, which is scale-independent, also shows a significant relationship with puncture performance, suggesting that these comparisons will hold at biological sizes. This is consistent with previous work on snake fangs illustrating that tip angle may be a better predictor of puncture performance than radius of curvature [34]. Furthermore, just because these spines may be able to puncture at their biological size, does not mean they can puncture particularly well. The small size means their actual depth of penetration, and subsequent anchoring ability, would be hindered. Overall, scaling in puncture systems is a complex issue that requires a great deal more research, and our results here illustrate that while disentangling the influence of scale and shape is difficult, there are many insights to be gained.

In addition to the altered scales, our tested spines were 3D-printed models derived from CT scans. While the material used to 3D-print the models has a tensile modulus (2.8 GPa) close to that of biological structures (e.g. 1.49 GPa [48]), it probably performs slightly differently than actual calcified structures.

We also assumed that most of the rotational movements of the spine would occur in the local sagittal plane, due to known hemipenial eversion mechanics, and thus only examined puncture thresholds within that single plane. However, it is possible that spines may rotate along other planes, and future work will need to examine the performance of the spines at other angles.

## Data Availability

Data and code for all analyses are available on Zenodo [49]. Supplementary material is available online [50].
